# Temporal variation in the prokaryotic community of a nearshore marine environment

**DOI:** 10.1038/s41598-022-20954-6

**Published:** 2022-10-07

**Authors:** Marino Korlević, Marsej Markovski, Gerhard J. Herndl, Mirjana Najdek

**Affiliations:** 1 Center for Marine Research, Ruđer Bošković Institute, Rovinj, Croatia; 2 Department of Functional and Evolutionary Ecology, University of Vienna, Vienna, Austria; 3 Department of Marine Microbiology and Biogeochemistry, Royal Netherlands Institute for Sea Research (NIOZ), Utrecht University, Den Burg, The Netherlands

**Keywords:** Microbial communities, Microbial ecology, Molecular ecology

## Abstract

Prokaryotic communities inhabiting surface waters of temperate areas exhibit patterns of seasonal succession. Generally, studies describing these temporal changes are not performed in the proximity to the coast. In the present study, temporal variation of these communities was determined in surface waters at two stations located in the close proximity to the eastern shore of the northern Adriatic Sea. Sequencing of the V4 region of the 16S rRNA gene identified the highest community richness in December with distinct shifts in community structure between periods from April to May, June to October, and November to March. Temperature was shown to be the main environmental force explaining community temporal variation. The NS5 marine group, uncultured *Cryomorphaceae*, SAR86 clade, and *Synechococcus* were present throughout the year. Members without know relatives within *Rhodobacteraceae* and the NS4 marine group were more pronounced in the period from April to May, the AEGEAN-169 marine group, SAR11 subclade III, and HIMB11 in the period from June to October, and SAR11 subclade Ia and *Archaea* in the period from November to March. *Litoricola* and OM60 (NOR5) clade were characteristic for both the community sampled from April to May and November to March. Taken together, prokaryotic communities inhabiting nearshore surface waters exhibit a general pattern in community structure similar to other surface associated assemblages of temperate areas. However, the identified specific community composition and temporal patterns differ from other coastal areas.

## Introduction

Prokaryotic picoplankton communities inhabiting marine surface waters exhibit seasonal succession. These temporal community changes were described for surface waters of polar, temperate, and (sub)tropical regions^1^. In temperate regions changes were mainly associated with summer water column stratification, winter mixing, and spring phytoplankton blooms^1–3^. Although general successional patterns in these waters have been reported, some local differences were also observed. While some studies have reported the exchange of multiple community states during the year^4–7^, others have observed a community separation in only two major groups, specifically the period from November to April (colder months) and from May to October (warmer months)^2^. Beside global patterns local conditions such as the presence and intensity of phytoplankton blooms^3,8,9^ or terrestrial nutrient inputs^10^ may influence seasonal community change.

Seasonal community sucession in temperate waters usually starts with assemblages characteristic for spring phytoplankton blooms. The successional pattern of different microbial groups during the pre-bloom, bloom, and bloom-decay periods have been described in detail^3,5,9^. The pre-bloom community is generally dominated by members of the alphaproteobacterial SAR11 clade, during the bloom taxa within *Bacteroidota*, such as *Formosa*, *Polaribacter*, *Ulvibacter*, and the VIS6 clade, become abundant, while the decay period is characterised by *Gammaproteobacteria*, i.e. the SAR92 clade^3,5,9^. Beside taxa co-occurring with phytoplankton blooms, communities specific to summer water stratification, characterised by the higher presence of *Flavobacteria* and *Synechococcus*, and communities specific for winter mixing, characterised by the higher relative abundance of the SAR11 clade, were described^2^. In addition, it was found that some subclades of SAR11 such as the subclade Ia are characteristic for summer and some such as subclades Ib and II for winter months^11^.

The majority of studies describing temporal changes in temperate areas were performed at long-term time series stations, such as the L4 sampling site of the Western Channel Observatory located in the Western English Channel^4,12^, Blanes Bay Microbial Observatory (BBMO) located in the northwestern Mediterranean^2,13^, Linnaeus Microbial Observatory located in the Baltic Sea^6^, station Kabeltonne in the German Bight (southeastern North Sea)^3,9^, and station E2 of the RADIALES time-series project located in the southern part of the Bay of Biscay^14^. All these stations, with the exception of station Kabeltonne, are located 0.5 nautical miles or more from the shore. In addition, seawater samples used in these studies were retrieved from surface waters. Data obtained from these time-series studies have found that a set of abiotic and biotic factors drive the temporal community variation^1^. It was suggested that biological interactions primarily affect microbial dynamics over time periods of days to weeks, while physicochemical parameters, such as light, temperature, and inorganic nutrient concentration, are mainly responsible for observed seasonal successional patterns^1,2,4,12,15,16^. In addition, several studies performed in surface waters of: station Kabeltonne, the San Pedro Ocean Time series location, and the Linnaeus Microbial Observatory indicate that phytoplankton derived dissolved organic matter (DOM) drives community dynamics^1,3,6,9,15^. It is therefore worth investigating whether such general interactions also apply to nearshore microbial communities in other areas such as the northern Adriatic Sea.

Surface waters at two stations along the eastern coast of the northern Adriatic Sea were sampled at monthly intervals to determine the temporal variation of prokaryotic picoplankton communities in these habitats. In addition, to assess the main environmental parameters associated with community change, compositional data were linked to a set of previously reported environmental parameters measured at the same time^17,18^.

## Results

Sequencing of 17 samples from the Bay of Saline and 18 samples from the Bay of Funtana (one of the samples was a sequencing replicate) yielded 1.5 million reads after quality curation and exclusion of sequences without known relatives (no relative sequences), eukaryotic, chloroplast, and mitochondrial sequences (Supplementary Table S1). The number of reads per sample ranged from 25,360 to 77,466 (Supplementary Fig. S1 and Table S1). Reads were clustered into 16,629 different OTUs at a similarity level of 97 %. To account for different sequencing depth reads were normalized to the minimum number of sequences per sample (25,360, Supplementary Table S1) that resulted in 13,440 different OTUs ranging from 608 to 1790 OTUs per sample (Supplementary Fig. S2).

Temporal variations in richness and diversity were determined by calculating the observed number of OTUs, Chao1 and ACE richness estimators, and Exponential Shannon and Inverse Simpson diversity indices. Similar trends in richness and diversity were observed at both stations (Supplementary Fig. S2) characterised by a maximum richness in both, the Saline (Observed Number of OTUs, 1790 OTUs) and Funtana (Observed Number of OTUs, 1786 OTUs) Bay in December 2017. In contrast, the Inverse Simpson index did not show an elevated value in December 2017 indicating that rare OTUs contributed substantially to the observed richness maxima. To determine temporal changes in the proportion of shared OTUs and communities the Jaccard’s and Bray-Curtis similarity coefficients were calculated between consecutive sampling points (Supplementary Fig. S3). Similar trends were observed at both stations with higher stability of shared bacterial and archaeal OTUs (Jaccard’s similarity coefficient) than shared communities (Bray-Curtis similarity coefficient). A substantial decline in community similarity between March and April 2018 was observed at both stations indicating a pronounced community shift in this period (Supplementary Fig. S3). Analysis of this time series data showed that only 0.6 % of OTUs were present throughout the study period, while these persistent OTUs contributed to 62.0 % of sequences. Taxonomic classification of these reads revealed that they mainly contributed to abundant phylogenetic groups described below (Supplementary Table S2).

**Figure 1:**
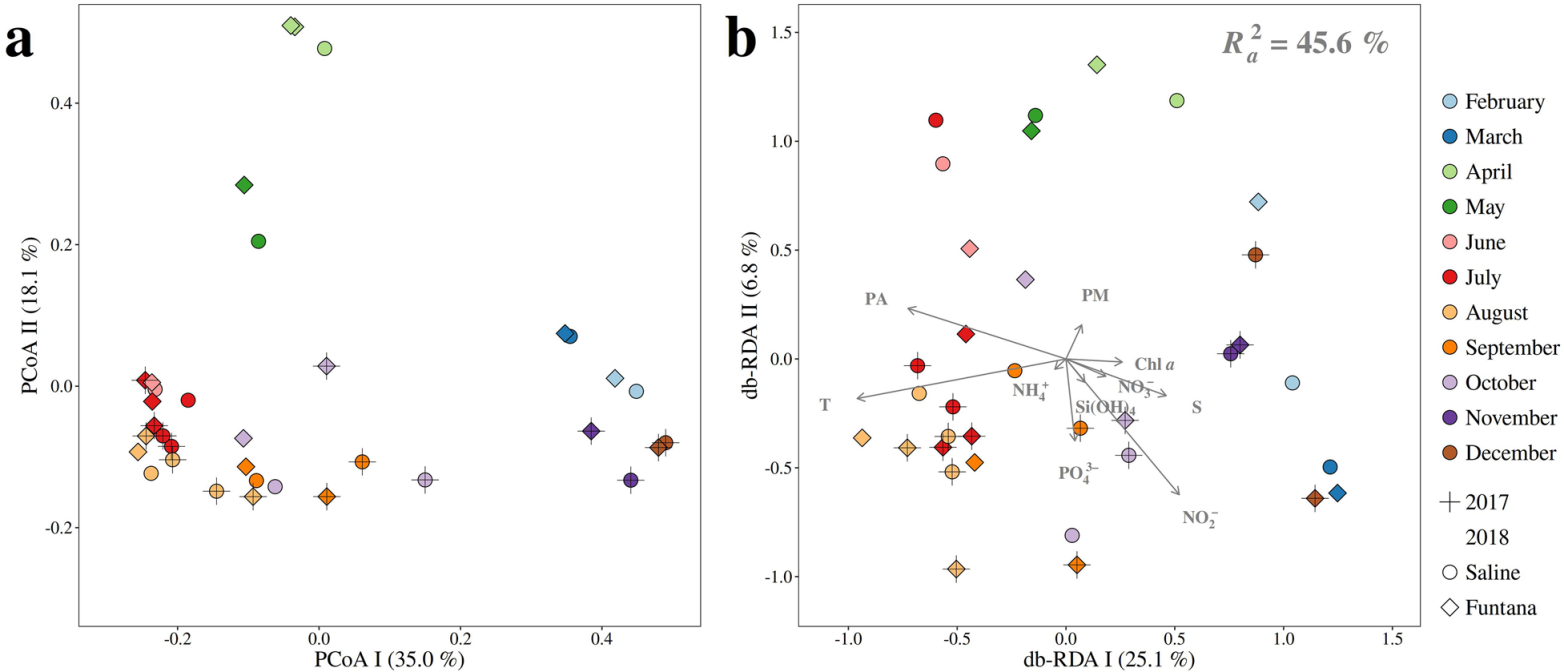
(**a**) Principal Coordinates Analysis (PCoA) of Bray-Curtis dissimilarities based on OTU abundances of bacterial and archaeal communities sampled in the Bay of Saline and Funtana. The proportion of explained variation by each axis is shown on the corresponding axis in parentheses. (**b**) Distance-based Redundancy Analysis (db-RDA) of Bray-Curtis dissimilarities based on the same community data sampled at the same locations and constrained by a set of environmental parameters (T – temperature, S – salinity, $\hbox {PO}_{4}^{3-}$ – orthophosphate, $\hbox {NH}_{4}^{+}$ – ammonium, $\hbox {NO}_{2}^{-}$ – nitrite, $\hbox {NO}_{3}^{-}$ – nitrate, $\hbox {Si(OH)}_{4}$ – silicic acid, PM – particulate matter, Chl *a* – chlorophyll *a*, and PA – prokaryotic abundance). Scaling type 2 and fitted site scores were selected to construct the plot. The proportion of community data variation explained by environmental variables ($R^2_a$) is stated on the biplot, while the proportion of community data variation explained by each canonical axis is shown on the corresponding axis in parenthesis. Samples in both plots originating from different months, years, and stations are labeled in different shape and color.

To evaluate the temporal variation of bacterial and archaeal communities Principal Coordinate Analysis (PCoA) was computed using Bray-Curtis dissimilarities based on OTU abundances (Fig. 1a). We identified regardless of the station sampled three separate communities: one specific for the period from June to October, one for the period from November to March, and one for the period from April to May. This separation into three specific communities was further supported by ANOSIM (R = 0.95, *P *< 0.01). To assess which environmental parameter mainly contributes to the observed temporal community variation, the community data were constrained by a set of environmental variables using distance-based Redundancy Analysis (db-RDA) (Fig. 1b). Nearly half ($R^2_a=$ 45.6 %) of the observed community variation could be explained by all the variables. Temperature, prokaryotic abundance, salinity, and nitrite mainly explained the separation between communities except for the community encompassing the period from April to May, whose separation could not be explained by any variable.

**Figure 2:**
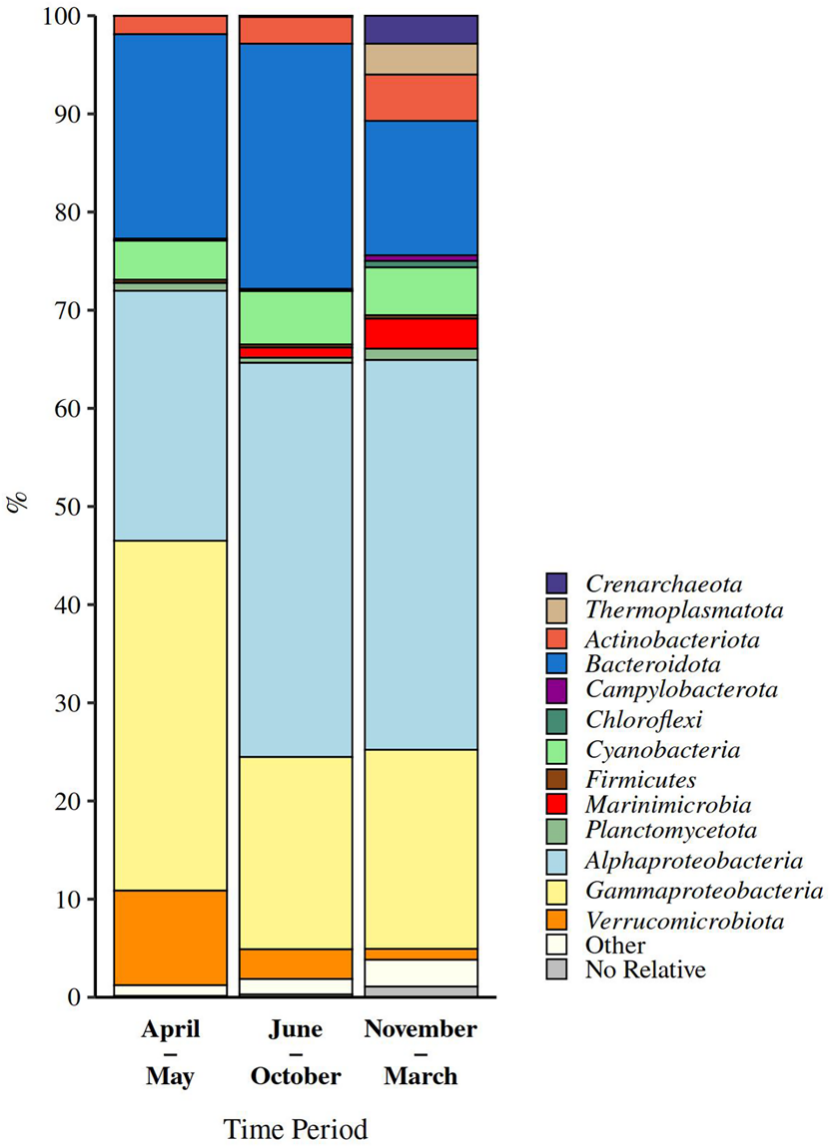
Taxonomic classification and relative contribution of the most abundant (≥ 1 %) bacterial and archaeal sequences during different time periods. No Relative – sequences without known relatives.

**Figure 3:**
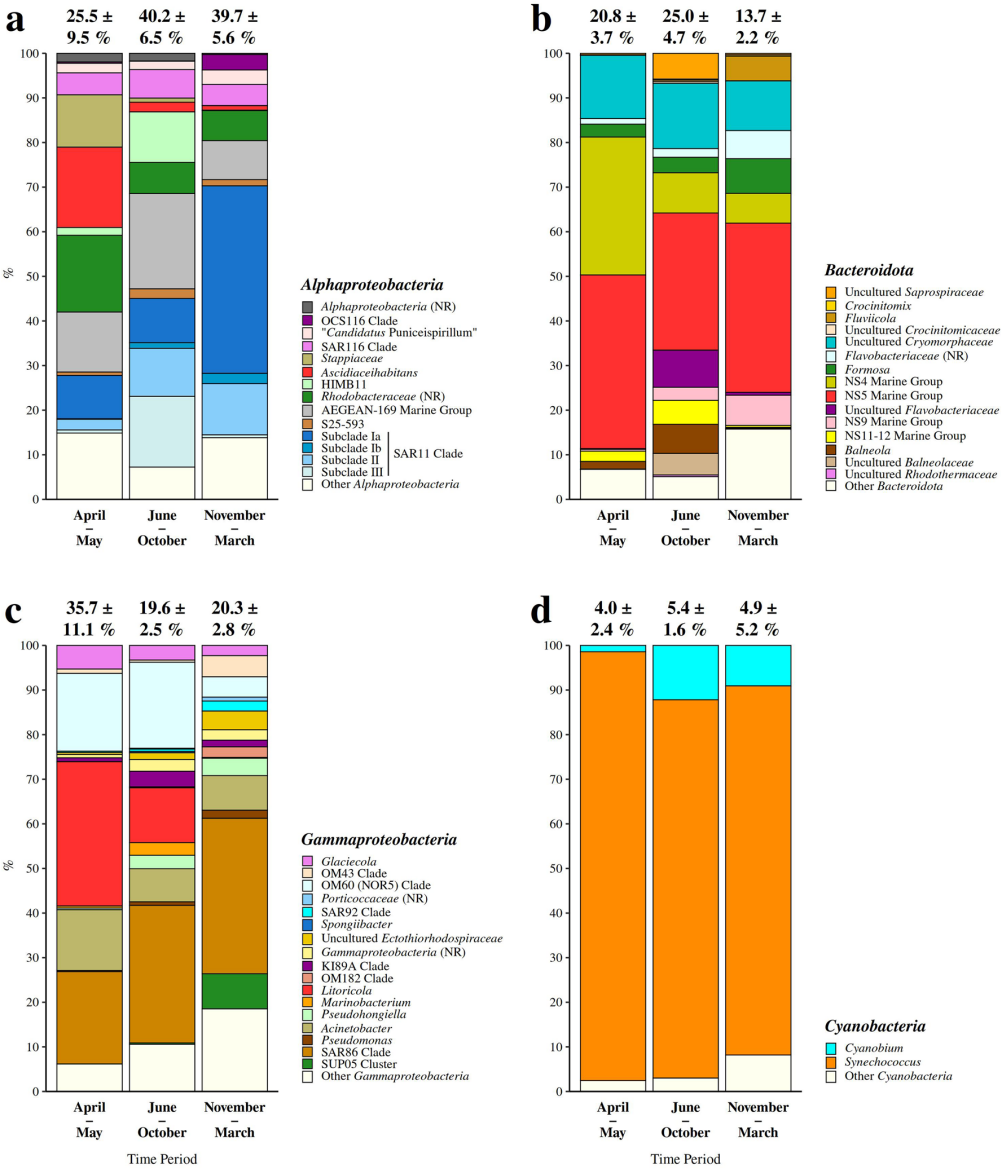
Taxonomic classification and relative contribution of the most abundant sequences within *Alphaproteobacteria* (≥ 2 %) (**a**), *Bacteroidota* (≥ 1 %) (**b**), *Gammaproteobacteria* ($\ge$ 1 %) (**c**), and *Cyanobacteria* (≥ 1 %) (d) during different time periods. The proportion of sequences classified into each of these taxa in the total bacterial and archaeal community is given above the corresponding bar. NR – No Relative (sequences without known relatives within the corresponding group).

The classification of reads showed that the prokaryotic community was dominated by bacterial (97.8 ± 3.8 %) over archaeal sequences (1.8 ± 3.5 %) (Fig. 2). The bacterial community was comprised of well-known seawater groups such as the *Actinobacteriota*, *Bacteroidota*, *Cyanobacteria*, *Marinimicrobia*, *Alphaproteobacteria*, *Gammaproteobacteria*, and *Verrucomicrobiota* (Fig. 2). Sequences classified as *Alphaproteobacteria* showed the highest relative abundance and comprised on average 38.3 ± 8.0 % of the prokaryotic community. The relative contribution of *Alphaproteobacteria* was higher in the period from June to October (40.2 ± 6.5 %) and November to March (39.7 ± 5.6 %) than in the period from April to May (25.5 ± 9.5 %) (Figs. 2 and 3a). Taxa within this group showed substantial variation between different temporal communities. The alphaproteobacterial community in the period from April to May was characterised by *Ascidiaceihabitans* (18.0 ± 6.8 %), no relative *Rhodobacteraceae* (17.2 ± 13.8 %), and *Stappiaceae* (11.7 ± 12.6 %), in the period from June to October by the AEGEAN-169 marine group (21.4 ± 7.7 %), SAR11 subclade III (15.9 ± 8.9 %), and HIMB11 (11.3 ± 5.1 %), while in the period from November to March by SAR11 subclade Ia (42.0 ± 4.7 %) (Fig. 3a).

*Bacteroidota* comprised on average 21.8 ± 6.2 % of the community. Higher values were found in the period from June to October (25.0 ± 4.7 %) and April to May (20.8 ± 3.7 %) than in the period from November to March (13.7 ± 2.2 %) (Figs. 2 and 3b). Some taxonomic groups within *Bacteroidota*, such as the NS5 marine group (30.7 ± 10.3 – 38.9 ± 11.4 %) and uncultured *Cryomorphaceae* (11.1 ± 5.1 – 14.6 ± 6.4 %), were characteristic for all the identified temporal communities, while others showed higher presence in one of the communities. For example, the NS4 marine group (30.9 ± 13.8 %) showed the highest contribution to the *Bacteroidota* community in the period from April to May, while uncultured *Flavobacteriaceae* (8.3 ± 6.3 %), *Balneola* (6.5 ± 3.8 %), the NS11-12 marine group (5.4 ± 1.7 %), and uncultured *Balneolaceae* (4.8 ± 4.0 %) were more pronounced in the period from June to October (Fig. 3b).

Reads classified as *Gammaproteobacteria* comprised on average 21.6 ± 6.6 % of the prokaryotic community. In contrast to *Alphaproteobacteria*, gammaproteobacterial sequences showed a higher relative abundance in the period from April to May (35.7 ± 11.1 %) than in other periods (June – October, 19.6 ± 2.5 % and November – March, 20.3 ± 2.8 %) (Figs. 2 and 3c). Within *Gammaproteobacteria* SAR86 clade was present throughout the study period (20.7 ± 10.5 – 34.8 ± 2.5 %). *Litoricola* and OM60 (NOR5) clade characterised the period from April to May (*Litoricola*, 32.4 ± 15.4 % and OM60 (NOR5) clade, 17.4 ± 2.3 %) and June to October (*Litoricola*, 12.3 ± 8.5 % and OM60 (NOR5) clade, 19.3 ± 5.1 %), while the SUP05 cluster was specific to the period from November to March (7.9 ± 4.2 %) (Fig. 3c).

*Cyanobacteria* comprised on average 5.1 ± 2.8 % of the total community. No large differences were found in the proportion of cyanobacterial sequences between the analysed periods (4.0 ± 2.4 – 5.4 ± 1.6 %) (Figs. 2 and 3d). Throughout the study period the cyanobacterial community was largely dominated by *Synechococcus* (82.7 ± 15.6 – 96.1 ± 3.0 %) (Fig. 3d). A higher relative contribution of archaeal reads was observed only in the period from November to March (6.4 ± 5.0 %), while in other periods their proportion in the total community was low (April – May, 0.2 ± 0.2 % and June – October, 0.4 ± 0.2 % (Fig. 2). The main taxonomic groups within *Archaea* contributing to the higher relative abundance of archaeal sequences in the period from November to March were the *Thermoplasmatota* Marine group II (56.2 ± 15.2 %) and *Crenarchaeota* “*Candidatus* Nitrosopumilus” (31.4 ± 20.8 %).

## Discussion

Prokaryotic communities inhabiting surface waters of polar, temperate, and (sub)tropical regions exhibit patterns of seasonal succession^1^. These temporal variations were mainly studied at long-term time series sites usually encompassing only one sampling station located further away from the coast^2,4^. In the present study the temporal variation of surface prokaryotic communities was determined at two sites in the close proximity of the shore.

Temporal changes in OTUs were considerable as indicated by the low proportion of OTUs present at each sampling date (0.6 %). This low number of persistent OTUs, however, comprised a high proportion of reads (62.0 %). Similar proportions of persistent core OTUs and their contribution to the total number of sequences were also reported in other time series studies^4,12^. Interestingly, the most abundant persistent OTUs were classified into taxa showing the highest proportion of reads. Analysis of the temporal variations in alpha-diversity showed maximal richness in December (Supplementary Fig. S2). This observation is in agreement with previously reported richness maxima in other regions during colder months^2,7,12,19^. It has been suggested that late autumn/winter overturn is responsible for this phenomenon by simply mixing populations from deeper parts of the water column with existing ones^1,11,20^. However, a similar richness pattern was also observed in regional seas where seasonal overturning of the water column does not play a role, such as in the shallow North Sea where also a higher richness was observed in winter^21^. Although the samples in this study were retrieved at very shallow locations, water column mixing taking place at deeper areas of the Adriatic Sea sustained with the circulation could bring additional taxa to these locations causing the observed increase in alpha-diversity.

We identified three distinct microbial assemblages characteristic for the period from April to May, June to October, and November to March (Fig. 1a). This is in agreement with studies describing the exchange of multiple communities over an annual cycle with a distinct spring community assemblage^6,7^ and in contrast to studies describing only a switch between winter- and summer-specific assemblages^2,8^. The distinct community detected in the period from April to May is likely a response to a phytoplankton bloom that can occur in this region^22,23^ as it was reported that the absence of spring and fall phytoplankton blooms in some areas can lead to a lower number of microbial assemblages^8^. Temperature and prokaryotic abundance were identified as main factors influencing the exchange of communities between the period from June to October and the period from November to March (Fig. 1b). It is not surprising that temperature and prokaryotic abundance almost equally explain this shift as higher prokaryotic abundances were reported in this area during summer months^24^. The identification of temperature as the single most important driver of community change is in line with previously reported data^2,7,8^. It was proposed that temperature indirectly influences community change through phytoplankton nutrient limitation during water column stratification and nutrient input in times of water column mixing^1^.

Taxonomic analysis revealed taxa characteristic for all the identified temporal communities, but also taxa characteristic for only one or two of the communities (Figs. 2 and 3). The flavobacterial NS5 marine group and uncultured *Cryomorphaceae*, the gammaproteobacterial SAR86 clade, and cyanobacterial *Synechococcus* were characteristic for all the identified communities. *Cryomorphaceae* are associated with organic matter re-mineralisation processes^25^, while a single-cell genome analysis of the NS5 marine group revealed its ability to degrade marine polysaccharides^26^. In addition, the NS5 marine group was previously detected in different seasons and environments of the Adriatic Sea^27,28^. These two groups could be a part of a basic re-mineralisation community present at this location throughout the year. The gammaproteobacterial SAR86 clade, previously reported in different environments of the Adriatic Sea^10,27,28^, was also detected in all communities. Recent analysis of metagenomic data suggests the existence of different functional and ecological ecotypes of this ubiquitous clade^29^. It is possible that different ecotypes are also characteristic for different seasons. The dominance of *Synechococcus* over other cyanobacterial groups in this coastal area was reported previously^10,30^. The known genome versatility of *Synechococcus* could explain the high contribution of this genus to the cyanobacterial community in fluctuating coastal environments^31^.

Differences between communities specific for the period from April to May, June to October, and November to March observed at the level of OTUs could also be seen in the taxonomic composition (Figs. 2 and 3). The identified community from April to May was characterised by the alphaproteobacterial *Ascidiaceihabitans*, no relative *Rhodobacteraceae*, and *Stappiaceae* and by the NS4 marine group from *Bacteroidota*. In addition to these groups characterising only the period from April to May the gammaproteobacterial OM60 (NOR5) clade and *Litoricola* were more pronounced in two periods, from April to May and from June to October. Members within the *Rhodobacteraceae* were previously associated with phytoplankton blooms in the North Sea^3,9^, while the NS4 marine group was found in studies describing bacterial communities in different environments of the Adriatic Sea with no clear association with increased autotrophic biomass^27,28^. The observed differences between the community characteristic for the period from April to May and spring-specific communities from other areas could therefore be explained by differences in structure and supply of phototroph-derived organic matter. The community originating from the period from June to October was characterised by the AEGEAN-169 marine group, SAR11 subclade III, and HIMB11 from *Alphaproteobacteria* and uncultured *Flavobacteriaceae*, the family *Balneolaceae* (*Balneola* and uncultured *Balneolaceae*), and the NS11-12 marine group from *Bacteroidota*. As mentioned above, this period was also characterised by the the OM60 (NOR5) clade and *Litoricola* from *Gammaproteobacteria*. A higher contribution of members such as the HIMB11 and the OM60 (NOR5) clade during the warm period of the year could result from their adaptation to more oligotrophic conditions during water column stratification through the ability to use alternative pathways of energy supply, e.g. bacteriochlorophyll and proteorhodopsin^32,33^. The period from November to March was characterised by the alphaproteobacterial SAR11 subclade Ia, the gammaproteobacterial SUP05 cluster, and the archaeal “*Candidatus* Nitrosopumilus” and Marine group II. In contrast to other studies^11,34,35^, we observed a higher contribution of the SAR11 subclade Ia in the colder period of the year. A study describing a strong co-dominance of “*Candidatus* Nitrosopumilus” and Marine group II suggests that nitrification by ammonia-oxidising archaea is coupled with ammonification performed by the members of the Marine group II^36^. In addition, the presence of “*Candidatus* Nitrosopumilus” reads in our samples is not surprising as recently two new strains of ammonia-oxidising archaea within the genus *Nitrosopumilus* have been isolated from northern Adriatic coastal waters^37^.

In conclusion, prokaryotic communities inhabiting the proximity of the shore exhibit temporal variations similar to surface water assemblages in other temperate regions. A richness maximum was recorded in the colder period of the year and temporal community shifts were observed with distinct community structures characteristic for periods from April to May, June to October, and November to March. Temperature was identified as the main force driving seasonal community change. Community compositions specific for the identified time periods and taxa exhibiting temporal patterns different from other coastal areas indicate that beside global driving factors local conditions also influence the coastal prokaryotic community.

## Methods

### Sampling

Seawater from the northern Adriatic Sea was collected by diving (depth,  1.5 m) in the proximity of the shore (25 – 50 m distance) in two bays  7 km apart, Saline ($$45^{\circ }$$$$7^{\prime }$$$$5^{\prime \prime }$$ N, $$13^{\circ }$$$$37^{\prime }$$$$20^{\prime \prime }$$ E) and Funtana ($$45^{\circ }$$ $$10^{\prime }$$$$39^{\prime \prime }$$ N, $$13^{\circ }$$$$35^{\prime }$$$$42^{\prime \prime }$$ E). The maximum depth at sampling stations was 3 – 4 m in the Bay of Saline and 2 – 2.5 m in the Bay of Funtana. Samples were collected in several 10 l containers and transported to the laboratory where 10 – 20 l were filtered through a 20 μm mesh. The filtrate was further sequentially filtered using a peristaltic pump through 3 and 0.2 μm polycarbonate membrane filters (Whatman, United Kingdom). Filters (0.2 μm) were dried briefly at room temperature and stored at −80 °C. Samples were collected monthly from July 2017 to October 2018. Concurrently of sampling for picoplankton community structure assessment, additional samples were collected to determine environmental parameters (temperature, salinity, orthophosphate, ammonium, nitrite, nitrate, silicic acid, particulate matter, chlorophyll *a*, and prokaryotic abundance) as reported previously^17,18^. Briefly, temperature and salinity were recorded on sampling dates by a pIONeer 65 probe (Radiometer Analytical, Denmark). Concentrations of orthophosphate, ammonium, nitrite, nitrate, and silicic acid were determined spectrophotometrically according to Strickland and Parsons (1972)^38^. Particulate matter was assessed gravimetrically by filtering up to 5 l of seawater through preweighed GF/F filters (Whatman, United Kingdom) and subsequently reweighing the filters after drying at 60 °C. Chlorophyll *a* was measured fluorometrically after seawater filtration through a GF/F filter (Whatman, United Kingdom) and extraction from filter in 90 % acetone^39^. Seawater samples for prokaryotic abundance assessment were fixed with formaldehyde (final concentration 4 %) and stained with 4,6-diamidino-2-phenylindol (DAPI, final concentration 1 μg ml^−1^) for 10 min^40^. DAPI stained samples were filtered through 0.2 μm black polycarbonate membrane filters (Whatman, United Kingdom). Abundances of prokaryotic cells were calculated after cell counting under an epifluorescence microscope (Zeiss Axio Imager Z1, Germany).

### DNA extraction

Picoplankton DNA was extracted from 0.2 μm polycarbonate filters according to Massana et al. ^41^ with slight modifications. Following phenol-chloroform extractions, 1/10 of 3 M sodium acetate (pH 5.2) was added. DNA was precipitated by the addition of 1 volume of chilled isopropanol, by incubating the mixtures overnight at −20 °C , and by centrifuging at 20,000 $\times$ g and 4 °C for 21 min. Pellets were washed twice with 500 μl of chilled 70 % ethanol and centrifuged after each washing step at 20,000 $\times$ g and 4 °C for 5 min. Air-dried pellets were resuspended in 50 μl of deionized water.

### Illumina 16S rRNA sequencing

The V4 region of the gene for 16S rRNA was sequenced using the Illumina MiSeq platform as described previously^42^. A two-step PCR procedure was applied to amplify the target region. In the first PCR, primers 515F ($5'$-GTGYCAGCMGCCGCGGTAA-$3'$) and 806R ($5'$-GGACTACNVGGGTWTCTAAT-$3'$) from the Earth Microbiome Project (https://earthmicrobiome.org/protocols-and-standards/16s) were used^43–45^. A tag sequence was added to these primers on their $5'$ end. PCR products were purified and sent for Illumina MiSeq sequencing at IMGM Laboratories, Martinsried, Germany. Prior to sequencing at IMGM, adapter and sample-specific index sequences were incorporated during the second PCR amplification of the two-step PCR procedure using primers targeting the tagged region. Beside samples, a positive and a negative control were included in each sequencing batch. For the positive control a mock community consisting of evenly mixed DNA material originating from 20 bacterial strains (ATCC MSA-1002, ATCC, USA) was used, while the negative control comprised PCR reactions without DNA template. Reads from this study (Bay of Saline) were combined with previously reported reads obtained using the same experimental procedure (Bay of Funtana)^46^ and analysed together. Previously reported reads were used in a study describing the temporal dynamics of epiphytic microbial communities on marine macrophyte surfaces solely to compare the epiphytic communities with assemblages from the surrounding ambient seawater^46^.

### Sequence and data analyses

Sequences obtained in the present study were analysed using mothur (version 1.43.0)^47^ according to the MiSeq Standard Operating Procedure (MiSeq SOP; https://mothur.org/wiki/MiSeq_SOP)^48^ and recommendations given by the Riffomonas project to enhance data reproducibility (https://riffomonas.org). Sequences were clustered into operational taxonomic units (OTUs) at a similarity level of 97 % as suggested by the MiSeq SOP. Computing was performed on the computer cluster Isabella (University Computing Centre, University of Zagreb). Alignment and classification were performed using the SILVA SSU Ref NR 99 database (release 138; https://www.arb-silva.de)^49,50^. The average sequencing error rate of 0.01 % was calculated based on the ATCC MSA-1002 mock community included in each sequencing batch, which is in line with previously reported values for next-generation 16S rRNA amplicon sequencing^48,51^. Also, negative controls processed together with the samples yielded only 2 sequences after quality curation.

Pipeline data processing and visualisation were done using R (version 4.1.1)^52^ in combination with packages vegan (version 2.5-7)^53^, stats (version 4.1.1)^52^, tidyverse (version 1.3.1)^54^, lemon (version 0.4.5)^55^, cowplot (version 1.1.1)^56^, RColorBrewer (version 1.1-2)^57^, kableExtra (version 1.3.4)^58^, rmarkdown (version 2.9)^59–61^, knitr (version 1.33)^62–64^, and tinytex (version 0.32)^65,66^. The observed number of OTUs, Chao1 and ACE richness estimators, and Shannon and Inverse Simpson diversity indices were calculated after normalization to the minimum number of reads per sample using vegan’s function rrarefy to account for different sequencing depths. Chao1 and ACE estimates were retrieved using vegan’s function estimateR, while Shannon and Inverse Simpson diversity indices were calculated using vegan’s function diversity^53^. To express both diversity indices in terms of effective number of OTUs the exponential of the Shannon diversity index was calculated^67^. The proportions of shared OTUs and communities between samples were expressed as the Jaccard’s (on presence/absence data) and Bray–Curtis similarity coefficient, respectively. Both coefficients were calculated on the OTU data table using vegan’s function vegdist and converted from dissimilarities to similarities^53,68,69^. Principal Coordinates Analysis (PCoA) was performed on Bray-Curtis dissimilarities based on OTU abundances using vegan’s function wcmdscale. The Lingoes correction method was applied to account for negative eigenvalues^53,69^. Difference between period-specific communities was tested by performing the Analysis of Similarities (ANOSIM) using vegan’s function anosim and 999 permutations^53^. Distance-based Redundancy Analysis (db-RDA) was computed by applying vegan’s function capscale to OTU abundances and explanatory environmental variables mentioned above. The analysis was performed on Bray-Curtis dissimilarities using the Lingoes correction for negative eigenvalues^53,68,69^. The proportion of community data variation explained by environmental variables ($R^2_a$) was computed using vegan’s function RsquareAdj^53,68^. Packages tidyverse, lemon, cowplot, and RColorBrewer were used for data analysis and visualisation, the stats package for general analysis, the help of package kableExtra to create tables, and packages rmarkdown, knitr, and tinytex to generate documents.

## Supplementary Information

Supplementary Information.

## Data Availability

The datasets generated and analysed during the current study are available in the European Nucleotide Archive (ENA) repository at EMBL-EBI under accession numbers SAMEA8117500 – SAMEA8117516, https://www.ebi.ac.uk/ena/browser/view/PRJEB43226, while the datasets analysed are available under accession numbers SAMEA6648771 – SAMEA6648788, SAMEA6648824, SAMEA6648825, https://www.ebi.ac.uk/ena/browser/view/PRJEB37267. The detailed analysis procedure including the R Markdown file are available in the GitHub repository (https://github.com/MicrobesRovinj/Korlevic_SeawaterDynamics_SciRep_2022).
