# Supplementary material for: Temporal variation in the prokaryotic community of a nearshore marine environment
Source: Sci Rep. 2022 Oct 7;12:16859. doi: 10.1038/s41598-022-20954-6 (PMC9547059; doi:10.1038/s41598-022-20954-6)
Supplement: Supplementary file 1 — Supplementary Information. [file 41598_2022_20954_MOESM1_ESM.pdf]

Supplementary Information

Temporal variation in the prokaryotic community of a nearshore marine environment

Marino Korlević[1*], Marsej Markovski[1], Gerhard J. Herndl[2,3], and Mirjana Najdek[1]

1. Center for Marine Research, Ruđer Bošković Institute, Croatia

2. Department of Functional and Evolutionary Ecology, University of Vienna, Austria

3. Department of Marine Microbiology and Biogeochemistry, Royal Netherlands Institute for Sea Research (NIOZ), Utrecht University, The Netherlands

[*]To whom correspondence should be addressed:

Marino Korlević

G. Paliaga 5, 52210 Rovinj, Croatia

Tel.: +385 52 804 768

Fax: +385 52 804 780

e-mail: marino.korlevic@irb.hr

Supplementary figures

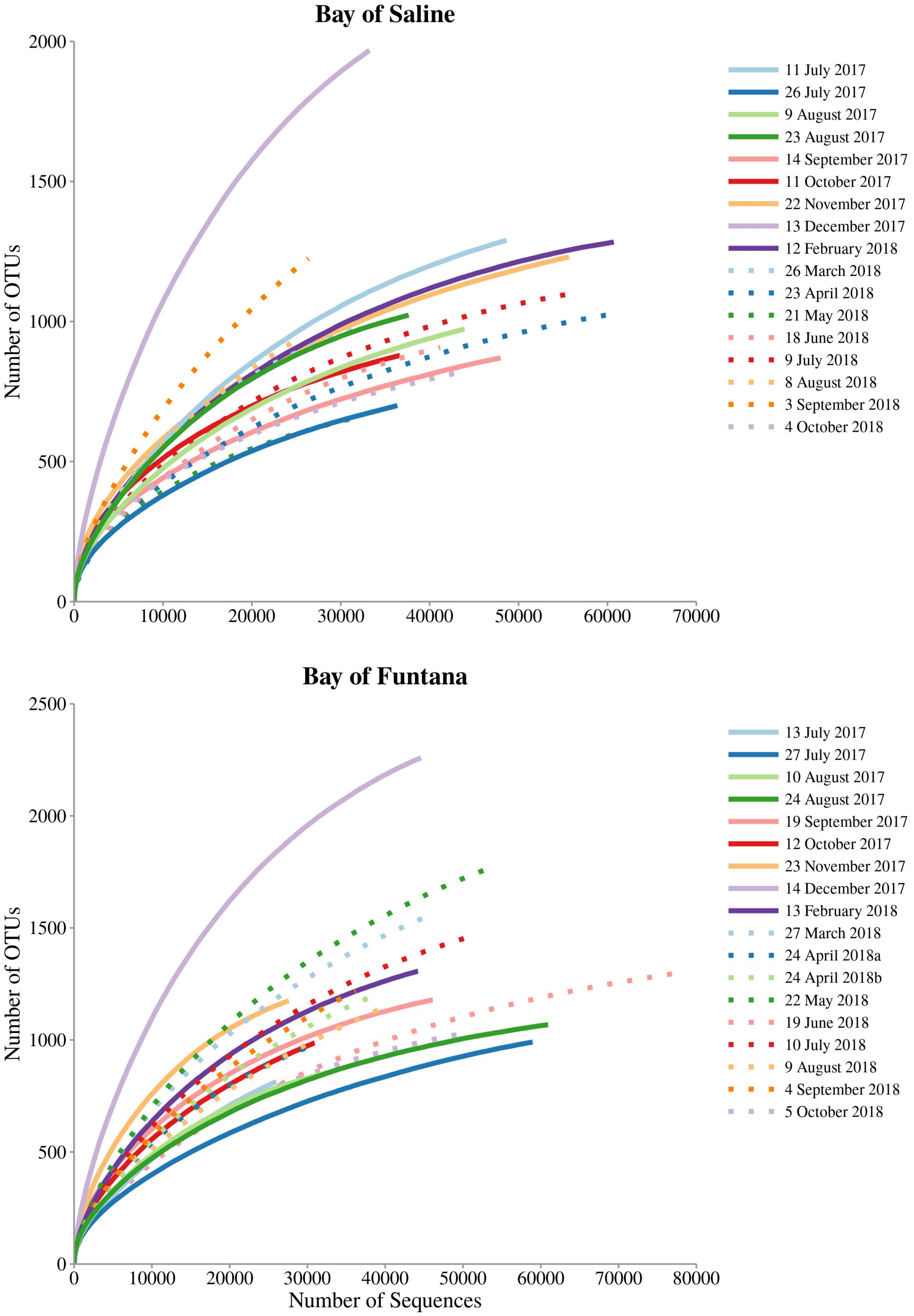

Supplementary Figure S1. Rarefaction curves of bacterial and archaeal communities sampled in the Bay of Saline and Funtana.

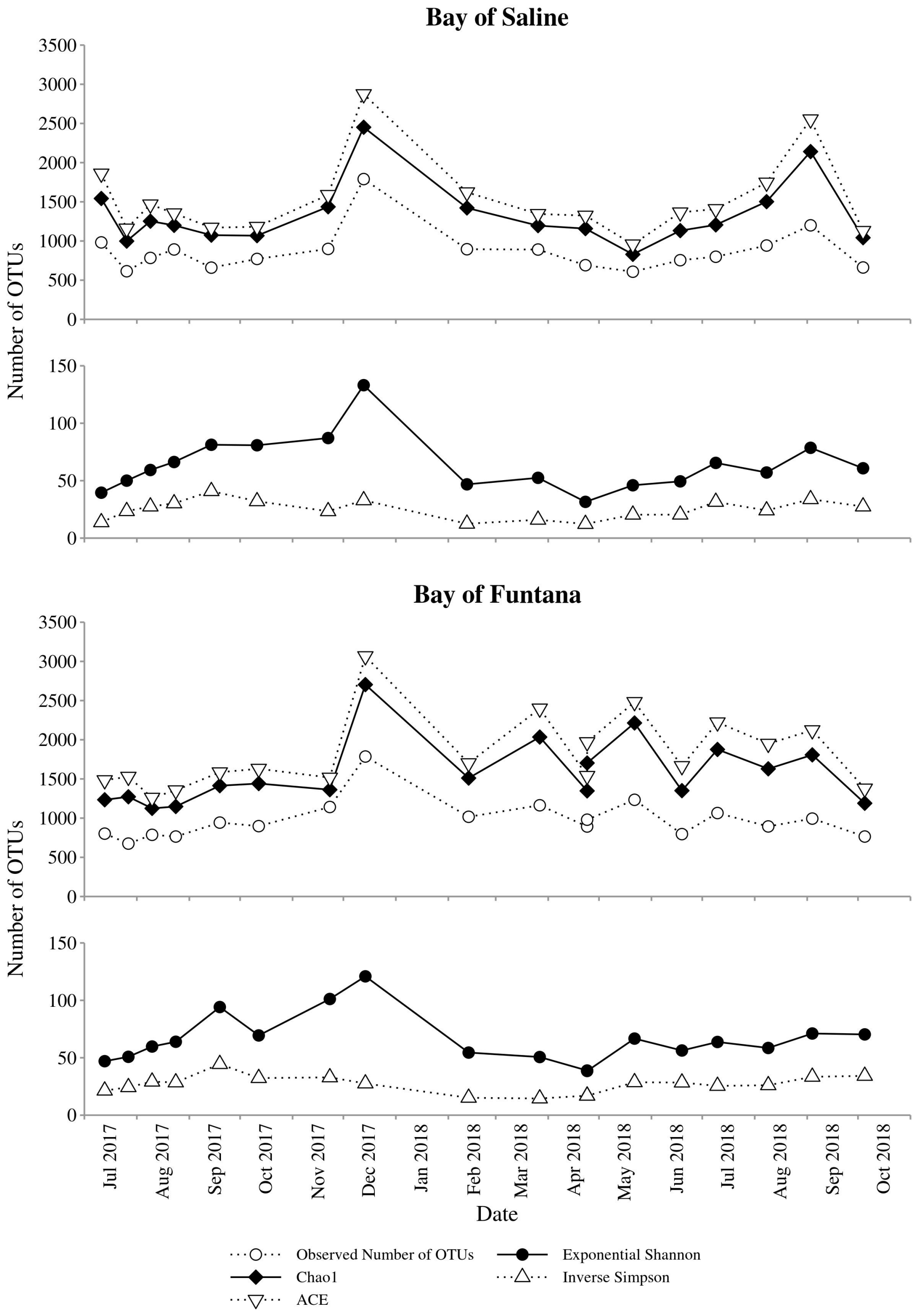

Supplementary Figure S2. Seasonal dynamics of observed number of OTUs, Chao1, ACE, exponential of the Shannon diversity index, and Inverse Simpson index of bacterial and archaeal communities sampled in the Bay of Saline and Funtana.

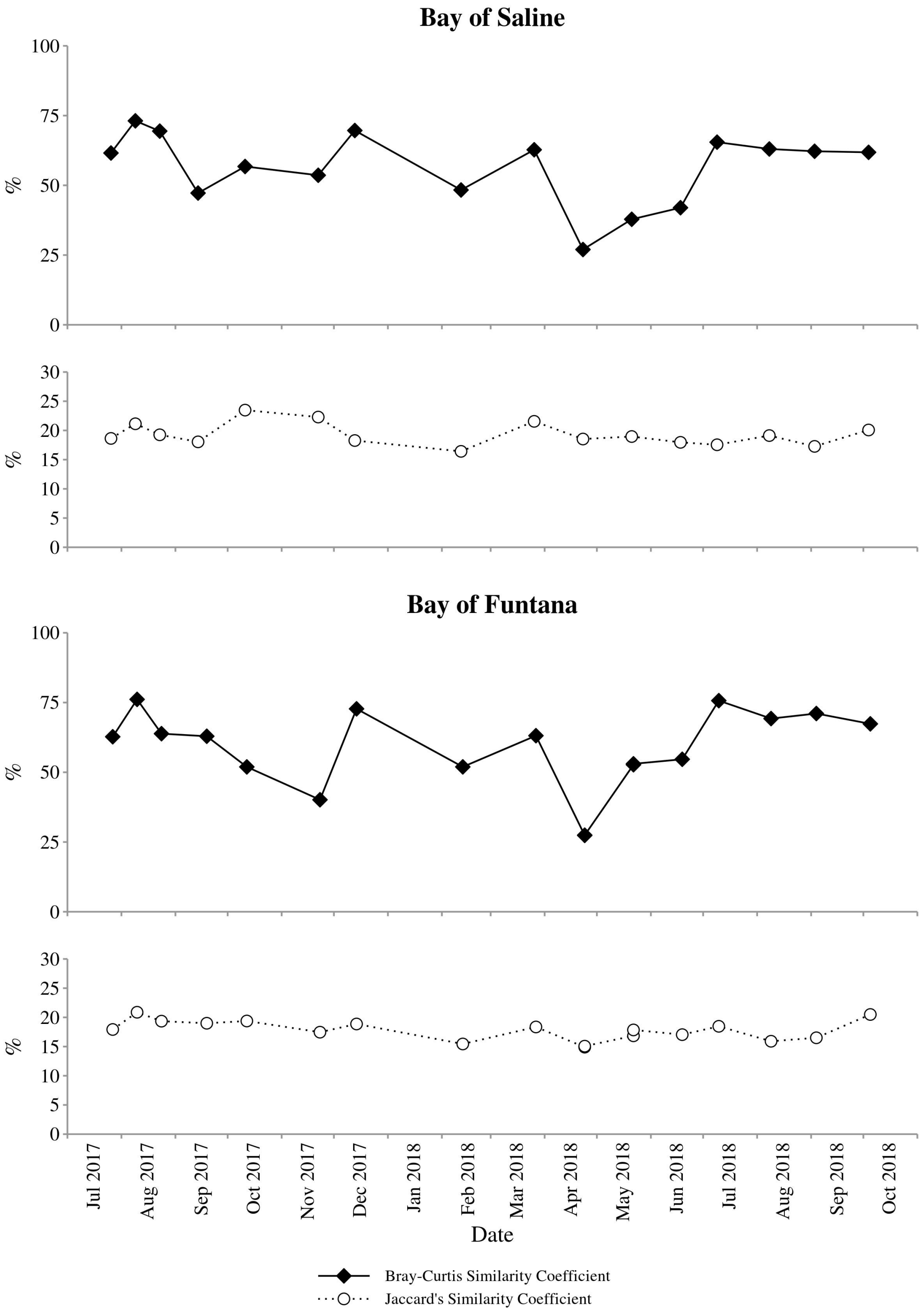

Supplementary Figure S3. Proportion of shared bacterial and archaeal communities (Bray-Curtis similarity coefficient) and shared bacterial and archaeal OTUs (Jaccard's similarity coefficient) between consecutive sampling dates of communities sampled in the Bay of Saline and Funtana.

Supplementary table

Supplementary Table S1. Sample ID, sampling station and date, and number of sequences and OTUs of each sample. The number of sequences and OTUs was calculated after exclusion of sequences without known relatives (no relative sequences) and eukaryotic, chloroplast, and mitochondrial sequences.

Sample ID	Station	Date	Number of Sequences	Number of OTUs
2	Bay of Saline	11 July 2017	48,652	1,290
3	Bay of Funtana	13 July 2017	25,989	814
4	Bay of Saline	26 July 2017	36,368	700
5	Bay of Funtana	27 July 2017	58,952	991
6	Bay of Saline	9 August 2017	43,934	973
7	Bay of Funtana	10 August 2017	32,624	855
8	Bay of Saline	23 August 2017	37,636	1,023
9	Bay of Funtana	24 August 2017	60,937	1,068
10	Bay of Saline	14 September 2017	47,977	870
11	Bay of Funtana	19 September 2017	46,110	1,179
12	Bay of Saline	11 October 2017	36,655	879
13	Bay of Funtana	12 October 2017	30,930	987
14	Bay of Saline	22 November 2017	55,678	1,231
15	Bay of Funtana	23 November 2017	27,586	1,175
16	Bay of Saline	13 December 2017	33,229	1,968
17	Bay of Funtana	14 December 2017	44,602	2,259
18	Bay of Saline	12 February 2018	60,714	1,284
19	Bay of Funtana	13 February 2018	44,215	1,307
20	Bay of Saline	26 March 2018	31,953	975
21	Bay of Funtana	27 March 2018	46,359	1,566
22	Bay of Saline	23 April 2018	60,343	1,026
23a	Bay of Funtana	24 April 2018	30,979	982
23b	Bay of Funtana	24 April 2018	38,573	1,200
24	Bay of Saline	21 May 2018	32,232	660
25	Bay of Funtana	22 May 2018	53,875	1,773
26	Bay of Saline	18 June 2018	41,180	908
27	Bay of Funtana	19 June 2018	77,466	1,298
28	Bay of Saline	9 July 2018	56,739	1,104
29	Bay of Funtana	10 July 2018	50,803	1,460

Supplementary Table S1. Sample ID, sampling station and date, and number of sequences and OTUs of each sample. The number of sequences and OTUs was calculated after exclusion of sequences without known relatives (no relative sequences) and eukaryotic, chloroplast, and mitochondrial sequences. *(continued)*

**Sample ID**	**Station**	**Date**	**Number of Sequences**	**Number of OTUs**
30	Bay of Saline	8 August 2018	25,360	942
31	Bay of Funtana	9 August 2018	39,058	1,133
32	Bay of Saline	3 September 2018	26,355	1,225
33	Bay of Funtana	4 September 2018	36,211	1,217
34	Bay of Saline	4 October 2018	43,955	823
35	Bay of Funtana	5 October 2018	49,573	1,026

Supplementary Table S2. Taxonomic classification of OTUs present at every sampling date at each station (Bay of Saline and Funtana). Only ten OTUs with the highest number of sequences after normalization to the minimum number of reads per sample are shown.

Station	OTU Number	No. of Sequences	OTU Taxonomy
Bay of Saline	Otu00001	33741	*Bacteria*; *Proteobacteria*; *Alphaproteobacteria*; SAR11 Clade; Subclade I; Subclade Ia
	Otu00002	27922	*Bacteria*; *Proteobacteria*; *Alphaproteobacteria*; *Rhodospirillales*; AEGEAN-169 Marine Group
	Otu00003	19674	*Bacteria*; *Cyanobacteria*; *Cyanobacteriia*; *Synechococcales*; *Cyanobiaceae*; *Synechococcus*
	Otu00004	19220	*Bacteria*; *Proteobacteria*; *Alphaproteobacteria*; SAR11 Clade; Subclade III
	Otu00005	17607	*Bacteria*; *Proteobacteria*; *Alphaproteobacteria*; SAR11 Clade; Subclade II
	Otu00006	16140	*Bacteria*; *Proteobacteria*; *Gammaproteobacteria*; SAR86 Clade
	Otu00007	12760	*Bacteria*; *Proteobacteria*; *Gammaproteobacteria*; *Oceanospirillales*; *Litoricolaceae*; *Litoricola*
	Otu00008	10799	*Bacteria*; *Bacteroidota*; *Bacteroidia*; *Flavobacteriales*; *Cryomorphaceae*; uncultured *Cryomorphaceae*
	Otu00011	9709	*Bacteria*; *Bacteroidota*; *Bacteroidia*; *Flavobacteriales*; *Flavobacteriaceae*; NS5 Marine Group
	Otu00012	7800	*Bacteria*; *Proteobacteria*; *Gammaproteobacteria*; *Cellvibrionales*; *Halieaceae*; OM60 Clade
Bay of Funtana	Otu00001	30081	*Bacteria*; *Proteobacteria*; *Alphaproteobacteria*; SAR11 Clade; Subclade I; Subclade Ia
	Otu00002	25323	*Bacteria*; *Proteobacteria*; *Alphaproteobacteria*; *Rhodospirillales*; AEGEAN-169 Marine Group
	Otu00003	20920	*Bacteria*; *Cyanobacteria*; *Cyanobacteriia*; *Synechococcales*; *Cyanobiaceae*; *Synechococcus*
	Otu00004	19157	*Bacteria*; *Proteobacteria*; *Alphaproteobacteria*; SAR11 Clade; Subclade III
	Otu00008	16312	*Bacteria*; *Bacteroidota*; *Bacteroidia*; *Flavobacteriales*; *Cryomorphaceae*; uncultured *Cryomorphaceae*
	Otu00005	16166	*Bacteria*; *Proteobacteria*; *Alphaproteobacteria*; SAR11 Clade; Subclade II
	Otu00010	16096	*Bacteria*; *Proteobacteria*; *Alphaproteobacteria*; *Rhodobacterales*; *Rhodobacteraceae*; *Ascidiaceihabitans*
	Otu00007	14847	*Bacteria*; *Proteobacteria*; *Gammaproteobacteria*; *Oceanospirillales*; *Litoricolaceae*; *Litoricola*
	Otu00006	12451	*Bacteria*; *Proteobacteria*; *Gammaproteobacteria*; SAR86 Clade
	Otu00011	9972	*Bacteria*; *Bacteroidota*; *Bacteroidia*; *Flavobacteriales*; *Flavobacteriaceae*; NS5 Marine Group
